# Event-Related Potential Correlates of Valence, Arousal, and Subjective Significance in Processing of an Emotional Stroop Task

**DOI:** 10.3389/fnhum.2021.617861

**Published:** 2021-02-25

**Authors:** Kamil K. Imbir, Joanna Duda-Goławska, Maciej Pastwa, Marta Jankowska, Jarosław Żygierewicz

**Affiliations:** ^1^Faculty of Psychology, University of Warsaw, Warsaw, Poland; ^2^Biomedical Physics Division, Institute of Experimental Physics, Faculty of Physics, University of Warsaw, Warsaw, Poland

**Keywords:** interference control, EST, factorial orthogonal manipulation ERPs, P2, N450

## Abstract

The present study is the first to measure event-related potentials associated with the processing of the emotional Stroop task (EST) with the use of an orthogonal factorial manipulation for emotional valence, arousal, and subjective significance (the importance of the current experience for goals and plans for the future). The current study aimed to investigate concurrently the role of the three dimensions describing the emotion-laden words for interference control measured in the classical version of the EST paradigm. The results showed that reaction times were affected by the emotional valence of presented words and the interactive effect of valence and arousal. The expected emotional arousal effect was only found in behavioral results for neutrally valenced words. Electrophysiological results showed valence and subjective significance correlated with the amplitude differences in the P2 component. Moreover, the amplitude of the N450 component varied with the level of subjective significance. This study also demonstrated that exploratory event-related potential analysis provides additional information beyond the classical component-based analysis. The obtained results show that cognitive control effects in the EST may be altered by manipulation in the subjective significance dimension.

## Introduction

The way we perceive environmental stimuli and interact with the surrounding world depends on our cognitive abilities. Cognitive control is especially important while planning behavior and executing actions. There are several aspects of environmental stimuli that may shape cognitive control capacity, including the way we perceive them in terms of emotional valence and arousal – in other words, the affective properties of stimuli (Russell, 2003). Apart from valence and arousal, the subjective significance has been proposed to be an additional factor important for cognitive control (Imbir, 2016d; Imbir et al., 2017a). The subjective significance is the activation-like aspect of emotional reaction that is analogical to the arousal but specific to the reflective mechanisms of processing (Strack and Deutsch, 2014), in other words, it is based on the propositional thinking and expresses the importance of the situation and thus the willingness of an individual to engage in an effortful mental processes, instead of using heuristics (Kahneman, 2011). This study examines the role of the above-listed three factors for performance in the emotional Stroop task (EST).

### Cognitive Control

Cognitive control is mental ability referring to mechanisms and processes that allow us to achieve our goals and focus on our plans instead of following automatic behavior, attentional biases, and involuntary actions (Gratton et al., 2018). Thus, this concept refers to the mechanisms of effortful processing in the reflective mind (Epstein, 2003; Strack and Deutsch, 2014). Among different forms of cognitive control, *interference control* seems to be especially interesting because (1) it refers to the competition of two easily defined processes responsible for reaction selection and (2) there is a simple task allowing for the assessment of this form of cognitive control, namely the Stroop task (Nigg, 2000; Cooper, 2010).

#### Paradigms for Measuring Cognitive Control

In the Stroop task (Stroop, 1935), a participant is asked to name the color of the font for presented words. There are two possible trials: congruent – the word meaning and the color of the ink are consistent (the word “green” is written in green) – and incongruent (the word “red” is written in blue). There are different results in terms of reaction time between congruent and incongruent trials that are evoked by the interference of the two processes engaged in solving the task: automated reading and controlled response to the task. The first one – reading the word and decoding the meaning – is an automatic and effortless process, following the long-lasting reading training that starts in childhood. The second one – naming the color of the ink – is an uncommon action that people rarely perform in everyday life, so the process is controlled and requires effort. The correct answer in the task is based on the controlled process; thus, the measured interference is high and shapes the reaction times (longer for incongruent compared with congruent trials).

The EST is based on the classic Stroop task (Stroop, 1935). In the EST, there are also two trials: incongruent and congruent. In this tool, some of the presented words are emotionally charged, and the interference is caused by the attentional bias toward emotional stimuli. It is more difficult to focus on the color naming task when attention is captured by the emotional meaning of the stimulus in comparison with the neutral word conditions. Both the classical Stroop test and the EST task require two different processes to appear: (1) inhibition of an automated action, which is reading the written word and decoding the semantic content, and (2) activation of a voluntary action that is part of the task – focusing on the color of the ink of the presented word (Larsen et al., 2006). Thus, the EST can be used to examine the interference between automatic and controlled processes in task solving.

#### The Impact of Emotional Factors on Cognitive Control

##### Valence

Valence may be most prominent among the emotional aspects of stimuli influencing the interference control measured in the EST (Kagan, 2007). The valence of a stimulus can be defined as the pleasantness versus unpleasantness of emotions evoked by external stimuli. Previous studies have shown that valence could influence performance in the EST (causing longer reaction times), mostly in the case of negatively valenced stimuli. That phenomenon was not observed for neutral words (Williams et al., 1996). In a study involving clinical patients with various traumatic experiences (McKenna and Sharma, 1995), the interference of negatively valenced stimuli on the EST performance was also observed when compared with neutral stimuli. However, regarding positive stimuli, such interferences were reported only if the positive stimuli were related to the concern of the traumatic experience (Riemann and McNally, 1995). Sharma and McKenna (2001) also noted that when the intervals between trials in a similar experiment involving valence were longer, the interference resulting from negative stimuli was significantly reduced. Furthermore, when stimuli were repeated, the impact of valence did not increase, as such repetition only led to habituation (McKenna and Sharma, 1995). Nevertheless, the authors of trauma-related studies have proposed further research in this direction: Emotional stimuli should be investigated not only in the dimension of valence, but also with the help of other factors, especially emotional arousal present in trauma experiences.

##### Arousal

Emotional arousal can be defined as an energy level of an organism that may be allocated to specific objects interpreted in an effective way (Russell, 2003). Arousal activates certain processes to cope with potentially fatal situations or engage in an appealing interaction with a potential sexual partner (Russell, 2003; Moors et al., 2013; Imbir et al., 2017a). The role of arousal is to activate mechanisms responsible for the appropriate reaction of individuals in the case of threatening or physically attractive stimuli – both important for survival – so it works on a highly automatic level (Kahneman, 2011). Epstein (2003) claims that arousal should be treated as an activation mechanism for simplified, heuristic, and effortless processing, which is specific to the so-called “experiential” mind (Epstein, 2003; Imbir, 2016a). Therefore, it is thought that arousal impairs higher processes, such as the above-mentioned cognitive control (Nigg, 2000), and it shifts the balance between simplified and complex mental processing in the direction of simplified thinking characteristic of the experiential mind (Epstein, 2003).

Researchers have confirmed the important role of arousal in the EST. Higher arousal is reported to make the reaction times longer (Burt, 2002; McKenna and Sharma, 2004; Imbir, 2016b). Previous studies indicate that a highly arousing stimulus, independently of the valence, causes longer reaction times in the color-naming task (Dresler et al., 2009; Frings et al., 2010). Frings et al. (2010), based on previous research (e.g., McKenna and Sharma, 2004), investigated the impact of the order of the valenced words on reaction time. However, the authors found that not only did valence cause a “slow” or “fast” effect in the EST, but arousal and relevance were responsible for the slowdown of reaction times in the EST. Dresler et al. (2009) found that arousing stimuli cause emotional interference for both positive and negative stimuli compared with neutral words. These results shed a different light on previous studies regarding the role of arousal in the EST, in which highly arousing stimuli elicited longer reaction times than words with a lower level of arousal, but the effect was more distinct for negative than positive stimuli (Pratto and John, 1991; Compton et al., 2003).

##### Subjective Significance

The aforementioned factors do not fully exhaust the complexity of affective processing. While it had been accepted that arousal activated effortless processing (Epstein, 2003), the mechanism activating controlled processing of complex mental actions specific to the rational mind remained unclear (Imbir, 2016a). Bearing in mind this gap, Imbir (2015) introduced the concept of subjective significance, which can be defined as an explicit attitude toward stimuli or events from the surroundings (Imbir, 2016a). Similarly to arousal, subjective significance results from the affective interpretation of environmental stimuli, but not the permanent trait of them (Russell, 2009). It is believed that the rational mind is based on congruent conceptual mechanisms. Therefore, it should have its own activation mechanism (Imbir, 2016c) that should work analogously as arousal. To put it simply, when a stimulus is treated as subjectively significant, it enhances the resources available for effortful rational mind processing. The subjective significance measurement can be performed using a Self-Assessment Manikin (SAM) scale proposed by Imbir (2015); it is analogous to the scale developed by Lang (1980) for arousal. The scale was introduced to participants to measure the bipolar dimension. It starts with experiences that are not subjectively significant to one’s goals, plans, and expectations. They could be referred to as trivial, gone, unnoticed, fleeting, inconsequential, insignificant, and unimportant. The scale ends with experiences that are very important to one’s goals, plans, and expectations. They could be referred to with words like vitally important, significant, turning-point, consequential, meaningful, and decisive. The scale appeared to be a reliable method – it provided stable and repeatable results – for assessing the subjective significance load of words (Imbir, 2015, 2016a), sentences (Imbir, 2016b, 2017b), and music pieces (Imbir and Goła̧b, 2017). In a recent study investigating the impact of arousal and subjective significance for modified performance in the EST (Imbir, 2016c), subjective significance reduced^1^ the slowdown of reaction times caused by the arousal. In the first experiment, participants performed a modified EST (with neutral words differing in activational properties of arousal and subjective significance), while in the second experiment, participants were asked to perform a classical Stroop test combined with the presentation of activation-laden words presented in black as distractors. In both experiments, the interaction between arousal and subjective significance was observed. The pattern of reaction time differences was that the highly arousing and moderately subjectively significant words group elicited longer reactions than both moderately arousing combined with moderately subjectively significant stimuli (arousal effect), as well as highly arousing stimuli combined with low and (or) high subjective significance.

Regarding the explanation of the pattern of reaction times presented above, we hypothesize that arousal activates automatic processes (reading the word, analyzing the meaning, and semantic content), which altogether makes the reaction time longer. Concurrently, subjective significance stimulates the controlled processes and increases the role of cognitive control, which results in shorter reaction time.

##### Non-emotional Factors

Aside from valence, arousal, and subjective significance, which are emotional factors, performance in the EST may be affected by word frequency, a purely linguistic factor. Burt (2002) reported that the reaction time of naming the color of high-frequency words was shorter than that of naming the color of low-frequency words. Further, Larsen et al. (2006) observed that negatively valenced emotionally laden words used in EST studies were typically both longer and lower in terms of frequency of occurrence than emotionally neutral words. In addition, the orthographic neighborhood of the control words was significantly less dense. Although valence and frequency have been confounded, one needs to note that the emotional Stroop effect was observed in experiments in which words did not differ in terms of frequency (Sutton and Altarriba, 2008). Thus, the EST phenomenon cannot be explained solely by word frequency (Kahan and Hely, 2008).

### ERP Correlates of Cognitive Control in EST

Cognitive control, as measured through the EST, has been the subject of interest in several different experiments combining behavioral and neurophysiological measures. Because reaction times are a good measure of cognitive load hindering processing, event-related potentials (ERPs) are considered to be indicators of certain task processing stages (both word processing and control of inhibition execution). Thus, ERP gives an insight into interference control at work, even when behavioral measures are not sensitive enough to show the effects (Thomas et al., 2007; Imbir et al., 2017b). We may expect to observe two main groups of ERP components:

(1) P2, and early posterior negativity (EPN), occurring during involuntary word reading and processing present in performance in the EST; and
(2) N450, late posterior negativity (LPN), and late positive component (LPC) – cognitive control and task interference-related effects present in the EST as well as word’s meaning connotations in the semantic network elicited by the word presentation and involuntary word meaning processing (van Hooff et al., 2008; Citron, 2012; Imbir et al., 2017a, b).

It has been suggested that the arousal effects would be observed within earlier components, while later components would be shaped by valence (Gianotti et al., 2008).

#### P2 Component

The P2 component, observed 200−250 ms after the stimuli onset, may be detected in centro-frontal and parieto-occipital areas, from which it is expected to originate (Freunberger et al., 2007). The component is related to threatening stimuli (which, in terms of dimensions, would be considered highly arousing negative stimuli), showing larger amplitudes than after neutral words (Thomas et al., 2007). When considering only the valence of emotions, electroencephalogram (EEG) results for this component seem inconsistent, showing larger amplitudes for positive (Schapkin et al., 2000), negative (Huang and Luo, 2006), or both positive and negative words (Carretié et al., 2004; Herbert et al., 2006) than for neutral ones. In a previous study (Imbir et al., 2017a), the amplitude of ERPs was susceptible to arousal and subjective significance differences of words in a 150−290-ms time window with a positive deflection of ERPs that was identified as the P2 component. The amplitudes were more positive for highly arousing stimuli than for less arousing words. Nevertheless, the amplitudes were larger for moderately significant stimuli than for highly significant words. This pattern of results closely resembles the pattern of differences in reaction times. This suggests that the P2 component reflects both involuntary word processing and decision-making in the EST (Imbir et al., 2017a).

#### EPN

Another early component related to word processing is EPN, occurring 200−300 ms after word presentation over the occipital scalp. It is considered to be an indicator of volitional attention (Citron, 2012). In this component, larger amplitudes may be observed for emotional words, either positive or negative, than for neutral ones, which may be understood as arousing versus non-arousing word division (Kissler et al., 2007; Herbert et al., 2008; Citron et al., 2013; Zhang et al., 2014).

#### N450

The first component clearly related to cognitive control during the EST is the N450, occurring in the time window of 350−500 ms, observed in fronto-central areas, sometimes also taking the shape of globally distributed negativity of an ERP (West and Alain, 2000; van Hooff et al., 2008). This component may be related to activation of the anterior cingulate cortex (Liotti et al., 2000), particularly the detection of conflicting characteristics of presented stimuli: Larger amplitudes occur in incongruent than in congruent trials (West and Alain, 1999; West, 2003; West et al., 2004). The ERPs of the N450 component vary depending on the valence of presented words: The amplitudes related to negative words are more negative than the neutral ones, which is in congruence with behavioral results, showing prolongation of reaction times for negative words (van Hooff et al., 2008). In the previous study (Imbir et al., 2017a), in which the authors utilized a modified EST for words differing in arousal and subjective significance, the authors observed effects within the N450 component. More negative amplitudes occurred for low subjectively significant words than for highly subjectively significant stimuli. Researchers have also found that this component was susceptible to the origin of an emotional state of words (automatic versus reflective, the factor postulated to represent the complexity of mechanisms underlying emotional reaction formation; Jarymowicz and Imbir, 2015). Amplitudes for stimuli of automatic origin were less negative than those for words of reflective origin or no specific origin (Imbir et al., 2017b).

Some authors have observed that the negative peak during stimuli processing occurs earlier; they have frequently labeled this phenomenon as the N400, which is also related to cognitive control. The N400 is a negative going deflection that peaks around 400 ms after the stimulus has occurred. The temporal range for this component is 200–600 ms and typically occurs in centro-parietal regions. This component is related to processing of the meaning of the stimulus, known from the discoveries of Kutas and Hillyard (1980). Despite the label, the potential is not always negative; sometimes, the deflection is more negative than in other conditions (Kutas and Federmeier, 2011). It is related to semantic deviations (incongruity) of words and other meaningful, non-verbal stimuli. Emotional state can also modulate the N400 amplitude in sentence processing (Federmeier et al., 2001). In that study, the authors observed the influence of mood for the N400 amplitude in sentence processing. In the case of a mildly positive mood, there were smaller amplitudes in the conditions of unexpected and distantly related words. This result indicates that semantic integration is facilitated in a positive mood. Other studies have confirmed findings about the emotional impact on this component (De Pascalis et al., 2009). Kiefer et al. (2007) found reduced N400 amplitudes for participants in a positive mood; the findings of Herbert et al. (2008) supported those observations. The N400 component could also be susceptible to novelty of the stimuli in a certain context, namely novel stimuli evoking greater amplitudes than the ones congruent with the context (Kutas and Hillyard, 1984; Kutas, 1993; St. George et al., 1997; van Berkum et al., 1999; De Pascalis et al., 2009). The mentioned experiments suggest that the emotional state of participants and properties of the stimuli – emotional and contextual – can influence the N400 amplitude.

#### LPC

The LPC occurs the latest, at 500–800 ms after stimulus onset in word processing in EST paradigms; it is distributed over parietal electrodes (Citron, 2012). The component is considered to be related to conscious recognition of the stimulus and its connotations, as well as further semantic processing (Hajcak et al., 2010; Stewart et al., 2010; Citron, 2012; Zhang et al., 2014). The amplitudes within this component may differ depending on whether the stimulus is threatening (Thomas et al., 2007) and related to reward, valence of the stimulus, and motivational engagement in the task (Citron, 2012). The results regarding the influence of valence on the shapes of the amplitudes of the LPC seem to be inconsistent. In earlier studies, researchers had reported more positive amplitudes for positive stimuli than for negative and neutral stimuli (Cuthbert et al., 2000; Herbert et al., 2006, 2008). In comparison, in newer reports, authors have shown more positive amplitudes for negative words than for neutral and positive ones (Kanske and Kotz, 2007; Hofmann et al., 2009; Schacht and Sommer, 2009; Gootjes et al., 2011). Other studies suggest that the general emotionality of the word influences the processing of the word within this component (Fields and Kuperberg, 2012; Citron et al., 2013; González-Villar et al., 2014). In our previous study, we found that the LPC was sensitive to the origin of the stimuli and words of reflective origin evoked more positive amplitudes than words of automatic origin (Imbir et al., 2017b).

### Hypotheses

We decided to employ for the first time an orthogonal manipulation of the valence, arousal, and subjective significance simultaneously, allowing us to study the main as well as interactive effects due to the emotional load of words on interference control effectiveness in the EST. This is an important advance in research because, in most cases, studies have been focused on a single factor or at most two factors at once. We also made an effort to align other important factors, such as the frequency of appearance and the length of words.

Given that the EST combines an automated process of word reading and involuntary access to word meaning with the controlled execution of a target action of font color naming, we may predict two types of effects. First, activation (arousal) accompanying emotions should disturb the controlled aspect of the EST and provide the activation for more automated action (word reading and further cognitive processing of a meaning); therefore, arousal should impair the inhibition control effectiveness and thus lengthen the time spent on responding and should affect the components associated with cognitive control. Second, we may also predict subjective significance plays a role that is opposite to arousal, giving an activation to the controlled aspect of the EST performance (untrained color of font naming), thereby reducing the arousal effect. Taking into consideration the above-mentioned argumentation and the results of earlier studies, we hypothesize for behavioral results that:

(H0) Valence does not affect reaction times in the EST.

(H1) The increasing levels of arousal increase the reaction times, that is, reaction times are longer for highly arousing stimuli compared with low and medium arousing stimuli.

(H2) The increasing levels of subjective significance decrease the reaction times, that is, reaction times are shorter for highly subjectively significant stimuli compared with low and medium subjectively significant stimuli.

(H3) Furthermore, there is an interactive effect between arousal and subjective significance, based on the fact that there is a reduction of reaction times for highly arousing stimuli with a high level of subjective significance. In other words, the longest reactions occur for stimuli of a high arousal level and a low subjective significance level, while the shortest reactions occur for stimuli with a low arousal level but high subjective significance level.

Considering the ERP correlates of EST performance, we hypothesize that (H4) the P2 component reflects the pattern of behavioral differences, as observed in earlier studies using EST (Epstein, 2003; Thomas et al., 2007; Imbir et al., 2017a, b), that is, arousal and subjective significance effects occur in the ERP amplitude in accordance with behavioral differences identified with the following relationship: The longer the reaction times are, the more positive the P2 amplitude. We also expect (H5) that the N450 component is susceptible to the detection of conflict in the control of interference (van Hooff et al., 2008; De Pascalis et al., 2009; Imbir et al., 2017a). Therefore, effects of subjective significance congruent with earlier findings, namely more negative amplitudes for low subjectively significant stimuli than for highly subjectively significant stimuli, are present for this component.

## Materials and Methods

### Participants

The participants were recruited from various faculties of Warsaw universities. They had to meet the following criteria to be included in the experimental group: they had to be right-handed native Polish speakers, without chronic clinical issues that may affect EEG recording directly (e.g., epilepsy) or because of the psychoactive drugs being taken. All of the participants had normal or corrected-to-normal vision. They received a small remuneration for taking part in the experiment.

Based on the effect sizes from previous studies involving similar procedures (Citron et al., 2013; González-Villar et al., 2014), we expected the eta squared (η^2^) for emotional effects on the EEG signals to range between 0.1 and 0.15. We conducted *a priori* sample size estimations using G-Power software (Faul et al., 2009), which showed that to achieve high power of the study at the level of 0.95 for the interaction of two factors, we would need at least 18 participants. Such a small sample size would be sufficient because of the design of the experiment, which involves a large number of repeated measures for each factor. We decided to double the estimated sample size to identify effects related to subjective significance, an emotional factor that had not been explored in the prior studies; thus, there could be smaller effect sizes than the ones used in estimations.

The experimental group comprised 36 subjects (18 men and 18 women), aged 19 to 35 years (M = 23.5, SD = 3.77 years). After collecting the data, five participants were excluded from EEG analyses because more than 50% of their trials had been rejected due to artifacts or extremely short or long reaction times. In the end, 31 participants were included in the additional analysis, 15 men and 16 women, aged between 19 and 28 years (M = 23.23, SD = 3.35 years).

We did not collect any personal data that would allow the participants to be identified. The participants provided informed consent to participate in the experiment, and this was documented in a research diary. The design, experimental conditions, and procedure were approved by the bioethical committee of the Faculty of Psychology at the University of Warsaw. All of the procedures involving human participants were conducted in accordance with the ethical standards of the institutional and/or national research committee, and with the 1964 Declaration of Helsinki and its later amendments or comparable ethical standards.

### Design

We investigated the behavioral and electrophysiological measures related to the reading of emotional words. We manipulated three factors – namely valence (three levels), arousal (three levels), and significance (three levels) – while controlling the following properties of words: frequency of appearance in language and length. The dependent variables were (1) reaction time in the EST and (2) amplitudes of ERPs for selected time windows or components.

### Materials

The words used in the study were acquired from the Affective Norms for Polish Words Reloaded database (Imbir, 2016a). Affective reactions to words have been measured on eight scales in the study comprising this database (including valence, arousal, and subjective significance). At least 50 participants (an equal number of men and women) provided measurements for each word on specially prepared Likert scales. The cohort of subjects scoring the words in the database was separate from those taking part in the current study. Their responses appeared to be reliable (in terms of split-half correlations as well as correlations of assessments to the previously introduced smaller word databases). Based on the collected ratings, the mean scores for each word on each of the eight scales were calculated.

To prepare stimuli for this experiment, we picked nouns with extreme values in valence (negative versus positive), arousal (low versus high), and subjective significance (low versus high) dimensions. The number of letters and the frequency of usage in the Polish language (Kazojć, 2011) were controlled among words to ensure the ecological validity of the manipulation. Words of no specific valence, arousal, or subjective significance were also employed as control groups (with their ratings between −0.5 SD and +0.5 SD around the mean on the particular scale). The experimental stimuli consisted of 27 groups (sets of words), 15 words in each group (for example, 15 positively valenced words, low on the arousal and subjective significance scale), with 405 words in total. The ratings on the experimental dimensions are tied in a specific way, for example, the highly arousing words are usually related to positive or negative emotions. Therefore, to specify the values of ratings for each of the word groups, we explored the data, determining the values on each dimension that could be considered low, medium, or high. We also report their statistics on the *z* scale calculated from all the nouns from the ANPW_R word database (Imbir, 2016a) to show how the means for the groups differ from the means in the entire database. The dimension of valence was divided into three levels, and the mean ratings for each of them were 3.98 (SD = 0.54; *z* = −0.88, SD(*z*) = 0.43) for negative, 5.12 (SD = 0.22; *z* = 0.02, SD(*z*) = 0.18) for neutral, and 6.15 (SD = 0.46; *z* = 0.83, SD(*z*) = 0.36) for positive words. Subsequently, for the dimension of arousal, the mean was 3.34 (SD = 0.26; *z* = −0.73, SD(*z*) = 0.29) for low arousal, 3.98 (SD = 0.15; *z* = −0.01, SD(*z*) = 0.17) for medium, and 4.75 (SD = 0.41; *z* = 0.86, SD(*z*) = 0.47) for highly arousing words. In the dimension of subjective significance, the mean rating was 3.01 (SD = 0.28; *z* = −0.72, SD(*z*) = 0.32) for words with low significance, 3.62 (SD = 0.14; *z* = −0.02, SD(*z*) = 0.16) for medium significance, and 4.36 (SD = 0.39; *z* = 0.83, SD(*z*) = 0.45) for the high significance group. All the words used in the experiment with their average ratings from the normative study on the three dimensions treated as experimental conditions and two other dimensions treated as control ones in this experiment can be found in Supplementary Appendix 1, sheet 1 (words).

The accuracy of selection was tested using analysis of variance (ANOVA) with a 3 (valence levels) × 3 (arousal levels) × 3 (subjective significance levels) model for all five dimension ratings (manipulated and controlled) treated as dependent variables. We expected to find effects of valence levels on valence ratings, arousal levels on arousal ratings, and subjective significance levels on subjective significance ratings, but no other effects. Such patterns of differences would support the validity of stimuli selection. We found significant differences between groups of different valence in the valence ratings [*F*(2, 378) = 892.62, *p* < 0.001, η^2^ = 0.83]. We did not find any significant differences between groups divided in terms of valence in any other experimental dimension, namely arousal [*F*(2, 378) = 0.68, *p* = 0.51, η^2^ = 0.01] or subjective significance of words [*F*(2, 378) = 2.28, *p* = 0.10, η^2^ = 0.01]. We did not find any differences either in the controlled dimensions of number of letters [*F*(2, 378) = 1.07, *p* = 0.34, η^2^ = 0.01] or the frequency of use in the Polish language [*F*(2, 378) = 0.65, *p* = 0.52, η^2^ = 0.01]. For the dimension of frequency of usage, we transformed raw data into natural logarithms, making the distribution of results closer to a normal distribution.

With regard to the groups divided by arousal, we found significant differences for arousal scale ratings [*F*(2, 378) = 767.77, *p* = 0.001, η^2^ = 0.80] but not for the scale of valence [*F*(2, 378) = 1.62, *p* = 0.20, η^2^ = 0.01] or subjective significance [*F*(2, 378) = 0.12, *p* = 0.89, η^2^ = 0.01]. There were also no differences between groups of different arousal levels in terms of word length [*F*(2, 378) = 0.06, *p* = 0.94, η^2^ = 0.01] or frequency of usage in language [*F*(2, 378) = 0.68, *p* = 0.51, η^2^ = 0.01].

With regard to groups of words divided by subjective significance, we found differences in the subjective significance scale ratings [*F*(2, 378) = 747.32, *p* < 0.001, η^2^ = 0.80], but not in the valence scale ratings [*F*(2, 378) = 0.72, *p* = 0.49, η^2^ = 0.01] or arousal ratings [*F*(2, 378) = 1.65, *p* = 0.19, η^2^ = 0.01]. We found no differences for the groups of different subjective significance in the number of letters [*F*(2, 378) = 2.60, *p* = 0.08, η^2^ = 0.01] or frequency of usage (*F*(2, 378) = 2.88, *p* = 0.06, η*^2^* = 0.02). Additional ANOVAs showed that there was no interaction effect for any of the three possible interactions of two experimental factors: Tests for valence and arousal, valence and subjective significance, and arousal and subjective significance turned out to be insignificant for the three experimental and two controlled dimensions. There were also no interaction effects for any of the three factors (valence, arousal, and subjective significance) on the three experimental scales, as well as the two controlled ones. All the results of the above-described ANOVAs may be found in Supplementary Appendix 1, sheet 2 (ANOVA).

To ensure that the experimental stimuli were correctly prepared, we also conducted ANOVAs within each of the experimental factors, treating each level of one factor as a different cluster of words. In other words, we checked differences on the three experimental scales and the two controlled ones on three levels of valence (negative, neutral, and positive), arousal (low, medium, and high), and subjective significance (low, medium, and high). All the results for these analyses can be found in Supplementary Appendix 1, respectively for valence, in sheet 3 (valence groups); arousal, in sheet 4 (arousal groups); and subjective significance, in sheet 5 (significance groups). Sheet 5 (Descriptive Statistics) in Supplementary Appendix 1 contains means and standard deviations for the three experimental and two controlled scales for all word groups, divided by the three experimental factors.

### Procedure

The experiment was conducted in an EEG laboratory. The subjects sat in a comfortable chair. The words were displayed on a 15.6-in. LCD screen at a distance of approximately 1 m from the subject’s eyes. The font was Helvetica 50-point size. Simultaneously with the target word, a cue (letters: P, C, Z, and N), indicating possible responses, was displayed underneath the target word on the screen. Each participant underwent a training session to learn what the task was and how to perform it correctly. The training consisted of 20 initial trials (naming colored squares displayed in one of the four target colors, reading color-meaning words) followed by the standard Stroop test (Stroop, 1935), that is, naming the font color of color-meaning words – both congruent and incongruent, presented in a random order. Participants were encouraged to respond as quickly and as accurately as possible. The subject’s task in the main part of the experiment was to indicate the font color of emotionally charged words.

**FIGURE 1:**
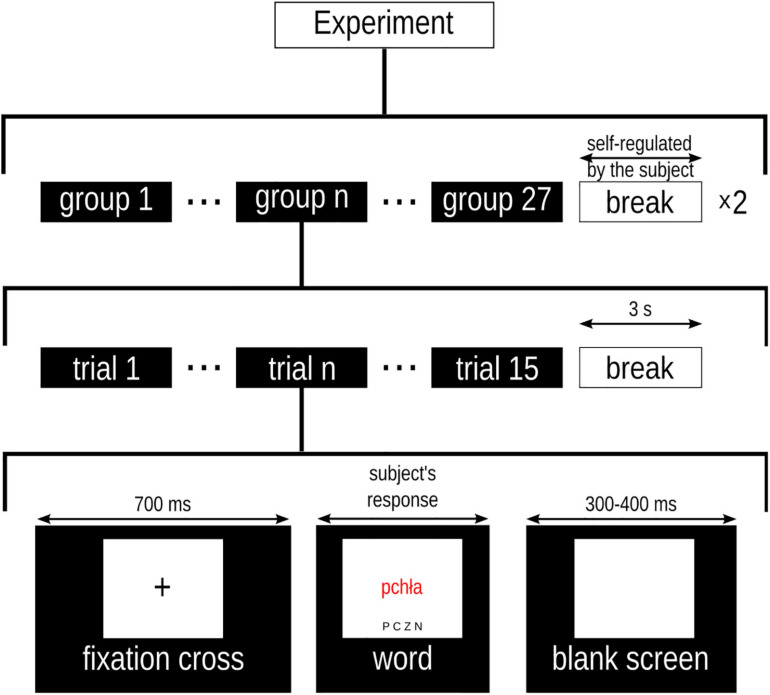
Outline of the experimental procedure.

The timing of a trial was as follows: a fixation cross was displayed for 700 ms, then a word was presented for as long as it took the subject to read and respond to it. The minimal stimulus presentation duration was set to 300 ms. Finally, the screen went blank for 350 ± 50 ms. The trials were grouped such that 15 words with homogeneous affective properties were presented consecutively. We decided on a block design because EST effects are more pronounced in this type of presentation (c.f. Bar-Haim et al., 2007). The subject could rest for 3 s after the presentation of each group. There were 27 groups in total, one for each possible combination of factor levels (3 valence × 3 significance × 3 arousal), comprising a list of 405 (27 × 15) words. The order of groups on the list and the order of words within each group were randomized. The experimental session had two parts separated by a longer break. The duration of the break was self-regulated by the subject. The experimental protocol is depicted in Figure 1.

### EEG Recording

#### Apparatus

The stimuli were displayed on a standard personal computer monitor. The stimuli were synchronized to EEG data by utilizing a circuit that recorded the changes in the brightness of a small rectangle on display, covered from the subject’s view. Its brightness changed synchronously with the content of the screen. We recorded the EEG signal from 19 electrode sites, namely Fz, Cz, Pz, Fp1/2, F7/8, F3/4, T7/8, C3/4, P7/P8, P3/4, and O1/2, referenced to linked earlobes. The ground electrode was placed at the AFz position. All impedances were kept at a similar value below 5 kOhm. The signal was acquired using a Porti7 (TMSI) amplifier, sampled at 2,048 Hz.

#### Offline EEG Signal Processing

We conducted the offline signal processing utilizing MATLAB^®^ with the EEGLAB toolbox (Delorme and Makeig, 2004) and custom-made scripts. The signal was zero-phase filtered.^2^ We used second-order Butterworth filters with 12 dB/octave roll-off; the high-pass filter cut-off was 0.1 Hz, and the low-pass cut-off was 30 Hz. Additionally, we used a notch filter for the 49.5sed a notch filter for the50.5 Hz band also implemented as the second-order Butterworth filter.

We extracted intervals ranging from -200 to 800 ms, with 0 being the onset of the target stimulus. The signals were baseline corrected to the interval -200 to 0 ms. We removed from further analysis trials that contained eye blinks, or in which the subject did not correctly identify the color of the presented word. The average error rate was 3.74%. We also removed trials with a reaction time shorter than the (Q1 − W) or longer than the (Q3 + W) of the logarithm of the reaction time individually for each subject, where Q1 is the 25th percentile, Q3 is the 75th percentile, and W = 1.5 × (Q3 − Q1). The reaction time for the analyzed data across all subjects is effectively within the range 287−3937 ms. Due to the extremely short or long reaction time, we had to exclude in total 2.71% of the trials (681 out of 31 × 810 = 25,100). The average number of trials per condition was 22.9 (SEM = 0.1), and it did not differ between the conditions.

### Statistical Analysis

The procedures were implemented in the R statistical package (R Development Core Team, 2017). The distribution of variables, response accuracy, and the number of correct and artifact-free trials were not Gaussian; therefore the significance of effects concerning these variables was assessed by means of the Friedman test for a replicated block design.

The effects concerning other variables, with approximately normal distributions, were assessed using ANOVA with repeated measures in a hierarchical procedure. We first analyzed the behavioral data and then the ERP data by using two different methodological approaches: an exploratory one and a classical one based on relevant EEG components suggested by the literature.

In the case of reaction times, we used logarithmic transformation to render the distribution normal. On the first level of analysis, a three-way ANOVA with repeated measures was applied. The transformed reaction time was the dependent variable and the valence, arousal, and significance were the independent ones. The significant main effects were analyzed with *post hoc* paired *t* tests with Holm’s correction for repeated comparison (Holm, 1979). On the second level of analysis, significant two-way interactions were further investigated by a series of one-way ANOVAs, with the levels of a selected variable set iteratively to subsequent levels. The selected variables were permuted. The significance of the effects repeatedly appearing in the series of ANOVAs was corrected for multiple comparisons by Bonferroni correction. The observed significant main effects, similarly to the first level, were further investigated using *post hoc t* tests with Holm’s correction. In this study, we did not obtain significant three-way interactions.

In the case of exploratory analysis of the EEG effects, there were two additional factors that we had to consider: time windows and regions of interest (ROIs). We performed a four-way ANOVA with repeated measures, one for each time window. The mean ERP amplitude within a given time window was the dependent variable, and the independent variables were valence, arousal, significance, and ROI. The significance of the effects repeatedly appearing in the series of ANOVAs was corrected for multiple comparisons by the Bonferroni correction. The significant main effects were analyzed with *post hoc* paired *t* tests with Holm’s correction. In this study, we did not obtain significant interaction effects in the exploratory analysis, so the investigation stopped at the first level.

The scheme of analysis of the classical EEG components was analogous to the one for the behavioral data. The dependent variable was the mean amplitude of a component (averaged across the electrodes and time range proper for the component), and the valence, arousal, and significance were the independent variables. We checked the sphericity with Mauchly’s test and applied the Greenhouse–Geisser correction where necessary.

All values (M and SEM) concerning time are expressed in milliseconds, and all values concerning amplitudes are expressed in microvolts.

## Results

### Behavioral Results

**FIGURE 2:**
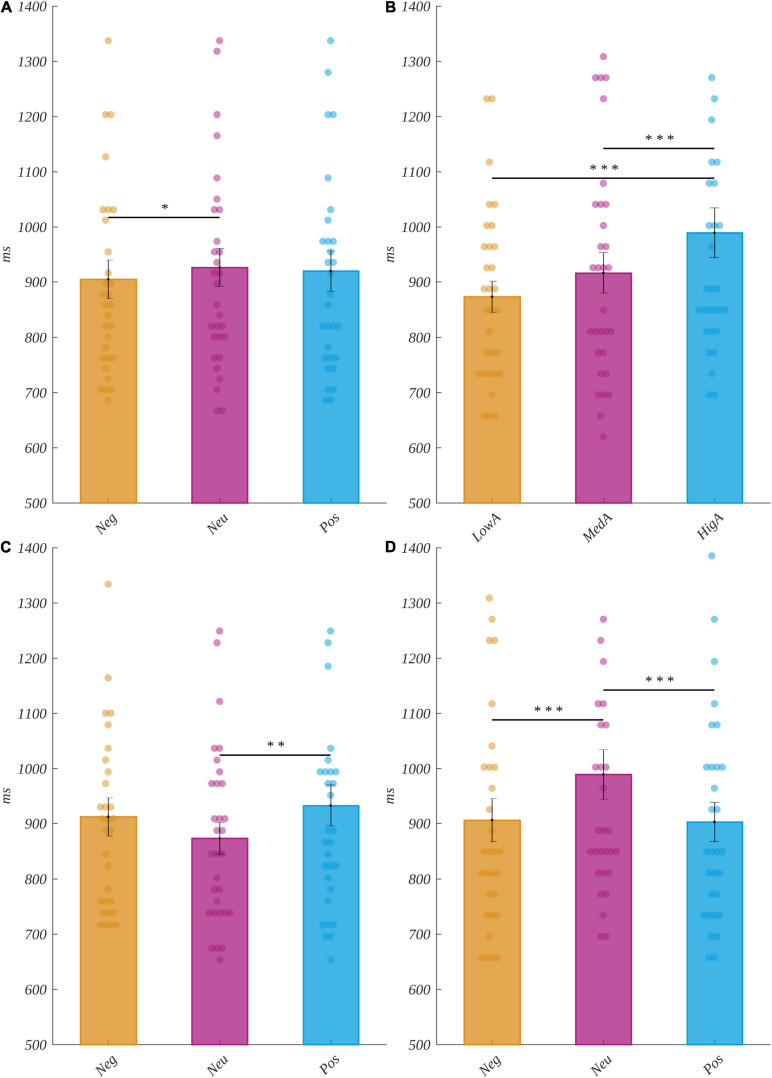
Reaction time effects. **(A)** The valence main effects. Interaction between valence and arousal: **(B)** differences between arousal levels for neutral words, and differences between valence levels for **(C)** low arousal and **(D)** high arousal words. Reaction times are in milliseconds. The bars represent the mean value, the error bars indicate the standard error of the mean (SEM), the individual dots mark the average amplitude of an individual subject in the given condition, and the asterisks indicate the significance level (*p < 0.05, **p < 0.01, ***p < 0.001, with Holm’s correction).

We analyzed the reaction times with a 3 (valence) × 3 (arousal) × 3 (subjective significance) repeated measures ANOVA. We obtained a significant valence main effect [*F*(2, 60) = 4.31, *p* = 0.02, η^2^ = 0.126]. The *post hoc* tests indicated that for neutral stimuli, the reaction time (M = 926, SEM = 34) was significantly longer than for negative ones (M = 905, SEM = 35; *t*(30) = 2.65, *p* = 0.04, *d* = 0.968). This relation is presented in Figure 2A. We did not observe significant differences between the levels of arousal [*F*(2, 60) = 3.25, *p* = 0.05, η^2^ = 0.098] or subjective significance [*F*(2, 60) = 2.09, *p* = 0.13, η^2^ = 0.065].

Furthermore, we observed an interaction between arousal and valence [*F*(4, 120) = 11.74, *p* < 0.001, η^2^ = 0.281]. The second-level ANOVAs showed that for neutral stimuli, there were differences in the reaction times due to the arousal level [*F*(2, 60) = 17.02, *p* < 0.001, η^2^ = 0.362]. Namely, for the high arousal level, the reaction times (M = 989, SEM = 45) were significantly longer than for both the medium arousal (M = 916, SEM = 36; *t*(30) = 4.28, *p* < 0.001, *d* = 1.564) and the low arousal (M = 873, SEM = 28; *t*(30) = 4.94, *p* < 0.001, *d* = 1.802) levels.

Moreover, the second-level ANOVAs indicated that for low arousing stimuli, the reaction time varied with the level of valence [*F*(2, 60) = 5.69, *p* = 0.005, η^2^ = 0.159]. Namely, it was longer for positive words (M = 933, SEM = 37) than for neutral ones (M = 873, SEM = 28; *t*(30) = 3.21, *p* = 0.01, *d* = 1.171). In addition, the reaction time in the case of highly arousing words varied with the level of valence [*F*(2, 60) = 22.89, *p* < 0.001, η^2^ = 0.433]. Here, for the neutral stimuli (M = 989, SEM = 45), it was longer than for both negative (M = 906, SEM = 39; *t*(30) = 3.94, *p* < 0.001, *d* = 1.439) and positive (M = 903, SEM = 35; *t*(30) = 5.77, *p* < 0.001, *d* = 2.107) stimuli. The interaction between valence and arousal is depicted in Figures 2B–D.

Neither the valence and subjective significance [*F*(4, 120) = 1.49, *p* = 0.21, η^2^ = 0.047] nor the arousal and subjective significance [*F*(4, 120) = 1.61, *p* = 0.18, η^2^ = 0.051] interactions were significant.

### ERP Exploratory Analysis

**FIGURE 3:**
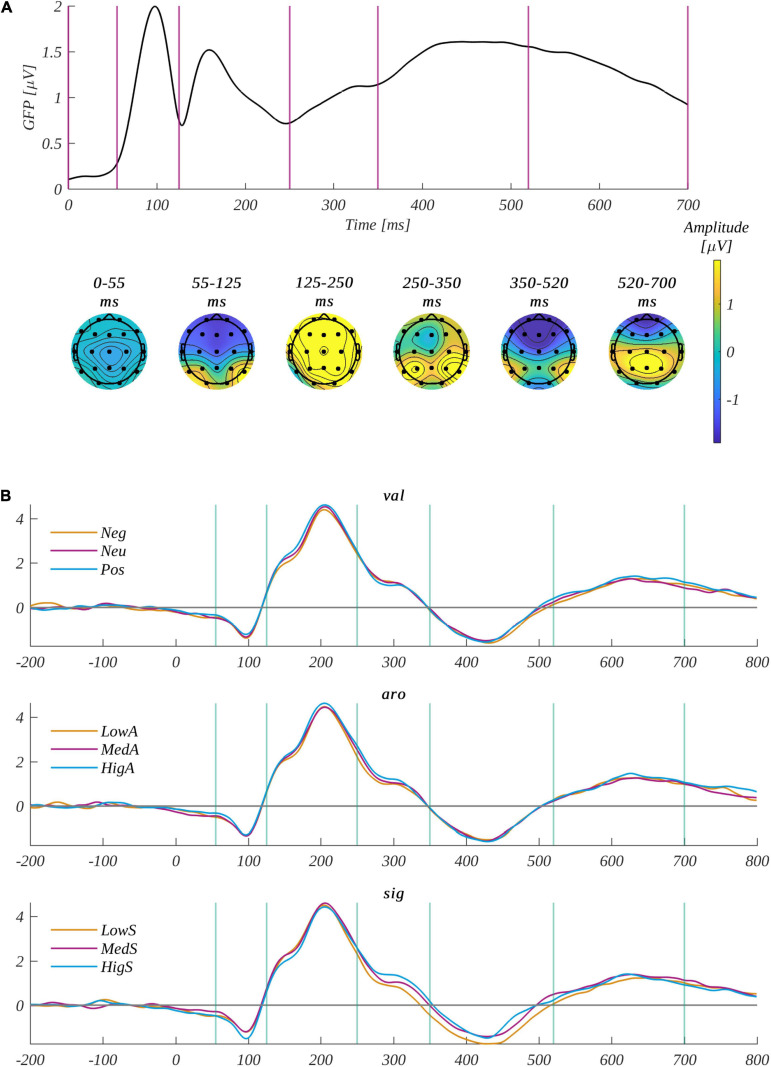
**(A)** Global field power (upper part) and topographies of average amplitude for a given time window (bottom part). The vertical lines in the upper plot indicate the time window boundaries. **(B)** The time course of electroencephalography (EEG) amplitudes averaged across all channels in the analyzed regions of interest (ROIs). The top subplot is for each level of valence, the middle subplot is for each level of arousal, and the bottom subplot is for each level of subjective significance. The vertical lines in the upper plot indicate the time window boundaries.

We conducted the exploratory analysis in five time windows: 55−125, 125−250, 250−350, 350−520, and 520−700 ms. We based this selection of the time ranges on the global field power (GFP) curve presented in Figure 3. We obtained the GFP as the standard deviation of the scalp potential across the electrodes at a given time (Lehmann and Skrandies, 1980; Skrandies, 1990). A local maximum of the GFP corresponds to a given distribution of electrical activity over the scalp (a microstate); a pass between the maxima in the curve corresponds to a reorganization of the distribution. Microstates corresponding to the selected time ranges are illustrated in the topographic plots of mean amplitude distribution at the bottom of Figure 3A.

We selected three ROIs: frontal (F: F3, Fz, F4), central (C: C3, Cz, C4), and parietal (P: P3, Pz, P4). We selected those ROIs to provide analysis comparable with our previous studies (Imbir et al., 2017a, b). Figure 3B illustrates the time course of averaged EEG amplitude for each level of the factors. We analyzed all the selected time windows using repeated-measures ANOVA, as described in the section “Statistical Analysis.” We only obtained significant effects in two of them, and these are reported below.

**FIGURE 4:**
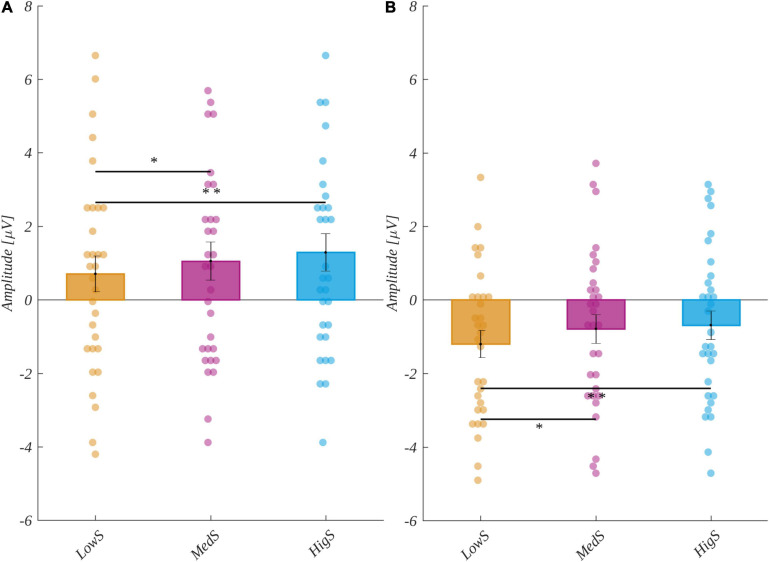
The exploratory event-related potential (ERP) analysis results: **(A)** for the 250−350 ms and **(B)** the 350−520 ms time windows. The bars represent the mean value, the error bars indicate the standard error of the mean (SEM), the individual dots mark the average amplitude of an individual subject in the given condition, and the asterisks indicate the significance level (*p < 0.05, **p < 0.01, ***p < 0.001, with Holm’s correction).

In the 250−350 ms time window, the subjective main effect was significant [*F*(2, 60) = 7.84, *p* = 0.001, η^2^ = 0.207]. *Post hoc* tests indicated that the amplitude for low subjectively significant stimuli (M = 0.7, SEM = 0.49) was less positive than for both high (M = 1.29, SEM = 0.51; *t*(30) = 3.50, *p* = 0.004, *d* = 1.279) and medium subjectively significant (M = 1.05, SEM = 0.52; *t*(30) = 2.76, *p* = 0.02, *d* = 1.009) ones (see Figure 4A). The main effects of valence [*F*(2, 60) = 0.02, *p* = 0.98, η^2^ = 0.001] and arousal [*F*(2, 60) = 0.73, *p* = 0.49, η^2^ = 0.024] were not statistically significant. Furthermore, none of the potential interactions were significant.

In the next time window (350−520 ms), the subjective main effect was significance [*F*(2, 60) = 7.74, *p* = 0.001, η^2^ = 0.205]. The ERP amplitude for low subjectively significant stimuli (M = −1.2, SEM = 0.37) was more negative than for both highly (M = −0.69, SEM = 0.38; *t*(30) = −3.96, *p* = 0.001, *d* = 1.446) and medium subjectively significant (M = −0.79, SEM = 0.39; *t*(30) = 2.89, *p* = 0.01, *d* = 1.054) ones (see Figure 4B). Neither the valence [*F*(2, 60) = 0.51, *p* = 0.60, η^2^ = 0.017] nor the arousal [*F*(2, 60) = 0.24, *p* = 0.79, η^2^ = 0.008] main effects nor the interactions were statistically significant. For completeness of the exploratory approach to the ERP analysis, we present in the Supplementary Appendix 2 the ERP time course for valence levels (Figure A1), subjective significance levels (Figure A2), and arousal levels (Figure A3) averaged across subjects for all recorded EEG channels.

### Classical Component-Based Analysis

To compare the results obtained using an exploratory approach with results based on component analysis, we performed an additional analysis for components typically susceptible to emotional properties of emotion-laden words in the EST, such as P2 and N450, that were found in an earlier study (Imbir et al., 2017b) to be related to arousal or subjective significance.

#### P2 Component

**FIGURE 5:**
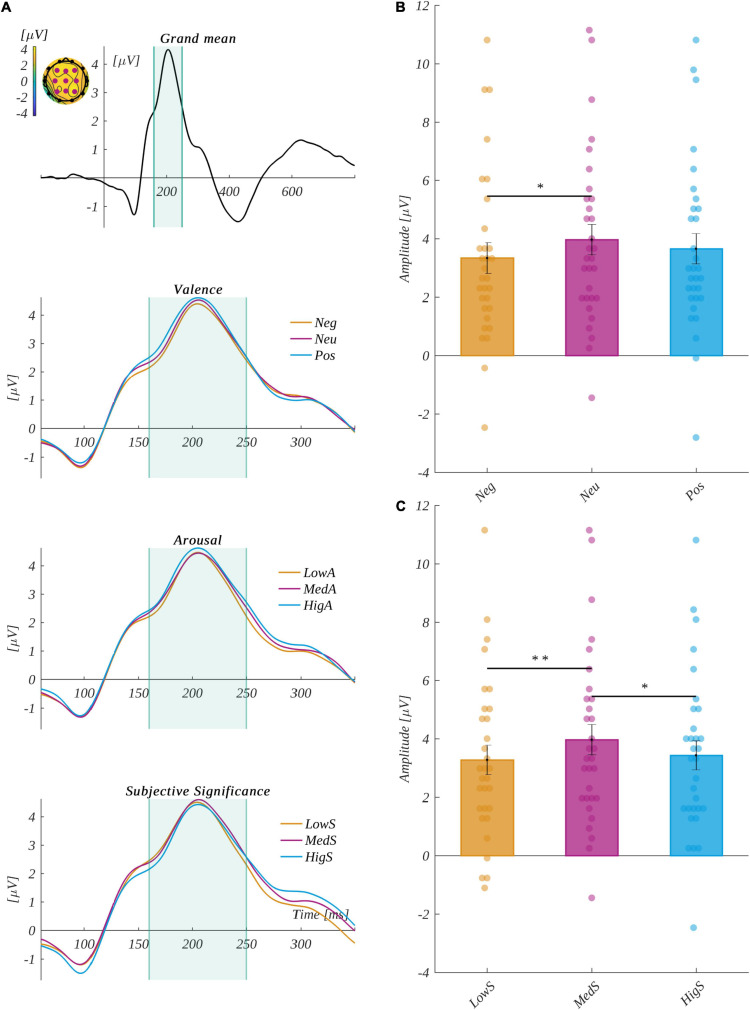
**(A)** The grand mean averaged across channels selected for component P2: P3, Pz, P4, C3, Cz, C4, F3, Fz, and F4. The vertical lines indicate the time range of component P2. The inset shows the topographical distribution of the potential in the selected time; the selected electrodes are marked. Below, the zooms on the interval surrounding the P2 component highlight each of the investigated factors. The top subplot is for each level of valence, the middle subplot is for each level of arousal, and the bottom subplot is for each level of subjective significance. Interaction between valence and subjective significance for the P2 component for: **(B)** medium significant words and **(C)** neutral words. The error bars mark the standard error of the mean (SEM). The significant differences are marked with black horizontal lines connecting respective levels. The asterisks indicate the significance level (*p < 0.05, **p < 0.01, ***p < 0.001, with Holm’s correction).

We defined the P2 component in this study as occurring across channels P3, Pz, P4, C3, Cz, C4, F3, Fz, and F4 within the 160−250-ms time range (Carretié et al., 2004; Huang and Luo, 2006). Figure 5A shows the time course of EEG amplitude averaged across the selected channels.

We analyzed the data with a 3 × 3 × 3 repeated-measures ANOVA. The valence [*F*(2, 60) = 2.75, *p* = 0.07, η^2^ = 0.084], arousal [*F*(2, 60) = 1.42, *p* = 0.25, η^2^ = 0.045], and subjective significance [*F*(2, 60) = 0.57, *p* = 0.57, η^2^ = 0.019] main effects were not statistically significant.

For the P2 component, there was a statistically significant interaction between valence and subjective significance [*F*(4, 120) = 3.10, *p* = 0.02, η^2^ = 0.094]. The second-level analysis revealed a significant valence main effect for medium subjectively significant stimuli [*F*(2, 60) = 5.55, *p* = 0.006, η^2^ = 0.156; see Figure 5B]. Here, *post hoc* tests showed that the amplitude for the neutral stimuli (M = 3.97, SEM = 0.52) was significantly more positive than for the negative ones (M = 3.33, SEM = 0.53; *t*(30) = 3.06, *p* = 0.02, *d* = 1.118).

We also observed a main effect of subjective significance for neutral stimuli [*F*(2, 60) = 6.47, *p* = 0.003, η^2^ = 0.177]. *Post hoc* tests showed that the amplitude for the medium subjectively significant words (M = 3.97, SEM = 0.52) was more positive than for both the highly subjectively significant (M = 3.43, SEM = 0.50; *t*(30) = 2.63, *p* = 0.026, *d* = 0.962) and the low subjectively significant (M = 3.28, SEM = 0.50; *t*(30) = 3.55, *p* = 0.004, *d* = 1.296) ones (see Figure 5C).

Neither the interaction between valence and arousal [*F*(4, 120) = 0.57, *p* = 0.69, η^2^ = 0.019] nor between arousal and subjective significance [*F*(4, 120) = 1.15, *p* = 0.33, η^2^ = 0.037] was significant.

#### N450 Component

**FIGURE 6:**
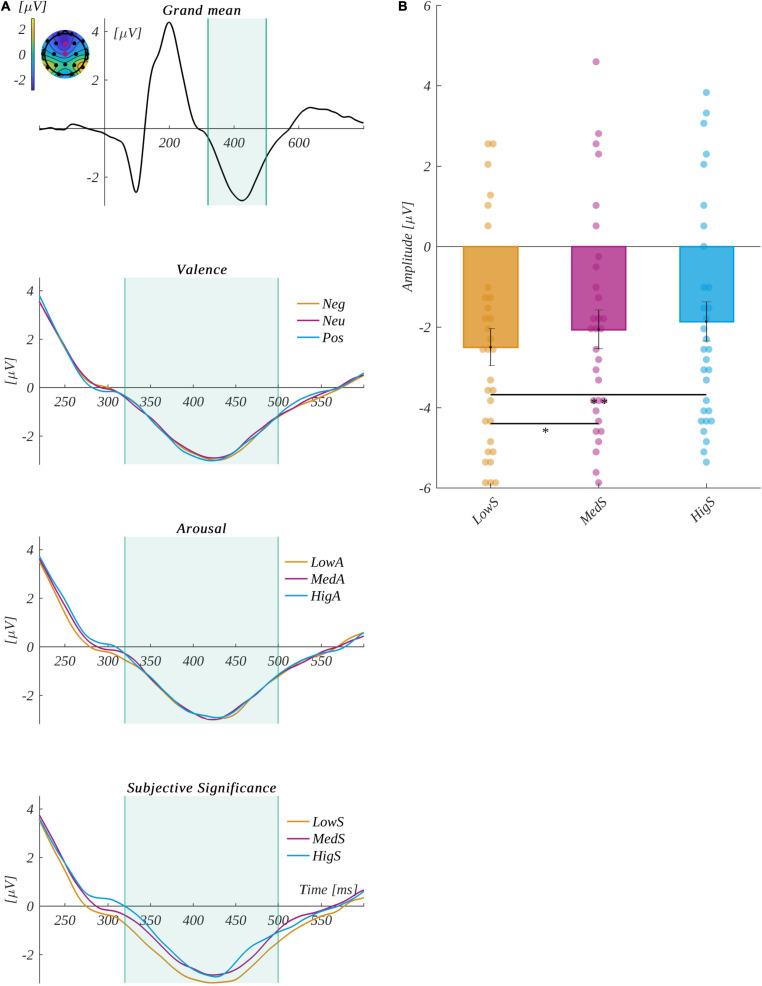
**(A)** The grand mean averaged across channels selected for component N450: Cz and Fz. The vertical lines indicate the time range of component P2. The inset shows the topographical distribution of the potential in the selected time; the selected electrodes are marked. Below, the zooms on the interval surrounding the N450 component highlight each of the investigated factors: each level of valence (top), arousal (middle), and subjective significance (bottom). **(B)** The main effects of subjective significance for the N450 component. The bars represent the mean value, the error bars indicate the standard error of the mean (SEM), the individual dots mark the average amplitude of an individual subject in the given condition, and the asterisks indicate the significance level (*p < 0.05, **p < 0.01, ***p < 0.001, with Holm’s correction).

We defined the N450 component in the current experiment as occurring at Cz and Fz electrodes in the time range from 320 to 500 ms (Liotti et al., 2000; West and Alain, 2000; van Hooff et al., 2008). The grand mean and the time course of the component for each level of the analyzed factors is plotted in Figure 6A.

ANOVA with repeated measures revealed differences between levels of subjective significance in the amplitude of the N450 component [*F*(2, 60) = 7.20, *p* = 0.002, η^2^ = 0.194]. The *post hoc* tests indicated that the amplitude for words of low significance (M = −2.50, SEM = 0.46) was more negative than for both highly significant (M = −1.86, SEM = 0.48; *t*(30) = 3.99, *p* = 0.001, *d* = 1.458) and medium significant (M = −2.06, SEM = 0.48; *t*(30) = 2.47, *p* = 0.04, *d* = 0.901) words. These relationships are presented in Figure 6B.

The other main effects and interactions were not statistically significant. Namely, we obtained the following statistical values for valence [*F*(2, 60) = 0.07, *p* = 0.93, η^2^ = 0.002], arousal [*F*(2, 60) = 0.05, *p* = 0.95, η^2^ = 0.002], the interaction between valence and arousal [*F*(4, 120) = 0.89, *p* = 0.47, η^2^ = 0.029], the interaction between valence and significance [*F*(4, 120) = 0.31, *p* = 0.87, η^2^ = 0.010], and the interaction between arousal and significance [*F*(4, 120) = 0.41, *p* = 0.80, η^2^ = 0.0.13].

## Discussion

The aim of the current study was to investigate simultaneously the effects of valence, arousal, and subjective significance on the behavioral performance and ERP correlates of the EST. Recent studies have identified that the slowdown in the EST is generated by the arousal level of stimuli rather than valence (Burt, 2002), but the effect of arousal in neutrally valenced stimuli was found to be modified by subjective significance (Imbir, 2015; Imbir et al., 2017a). The most important issue now is to check how the inclusion of subjective significance would influence valence and arousal effects in the EST.

### Behavioral Results

The main behavioral effect we observed was related to valence, namely longer reaction times for neutrally valenced words compared with the negative ones – an effect that we did not predict in our hypotheses. In general, the reverse pattern to the one observed in this study, that is, longer reaction times for negative stimuli, was present in early EST behavioral studies (c.f. McKenna and Sharma, 1995; Larsen et al., 2006), when there was no control for the arousal level of emotive categories. Further studies showed that valence effects were not present when emotive categories were aligned with arousal levels; instead, linear arousal effects appeared, showing longer reaction times for more arousing stimuli irrespective of their valence (e.g., McKenna and Sharma, 2004; Frings et al., 2010). Nevertheless, it is worth noting that in some of the current studies, a similar effect – that is, longer reaction times in the EST for neutral stimuli than for valenced stimuli – was observed for low arousing stimuli, but not for highly arousing ones (Feroz et al., 2017). In our current experiment, each valence category was aligned for arousal (negative words: M = 4.05, SD = 0.61; neutral words: M = 4.02, SD = 0.64; and positive words: M = 4, SD = 0.69, see Supplementary Appendix 1, spreadsheet 6) as well as frequency of appearance and length. These data support the validity of the obtained result.

We have also found the interaction between valence and arousal, which we partially predicted in our hypotheses. The validity of the paradigm is supported by the presence of the expected arousal effect in the group of neutrally valenced words. Highly arousing neutral words elicited longer reaction times than low arousing and medium arousing neutral stimuli. This outcome suggests that effects of arousal were clearly expressed when not accompanied by valence. This is in line with previous findings regarding the influence of arousal on cognitive control (Burt, 2002; McKenna and Sharma, 2004; Dresler et al., 2009; Frings et al., 2010; Imbir, 2016b). In the case of low arousing stimuli, we found that positive words elicited longer reaction times than neutral words. The obtained interaction also replicates the main effect of valence for highly arousing stimuli: Neutrally valenced stimuli elicited longer reaction times than negatively and positively valenced stimuli, which is in contrast to Feroz et al.’s (2017) behavioral results showing a similar valence effect for low arousing stimuli.

We did not observe the expected effect of subjective significance, or the interaction with valence or arousal. In our previous studies, which we have mentioned in broad terms in this article (Imbir, 2015; Imbir et al., 2017a), we observed behavioral effects of subjective significance – namely, the moderately significant stimuli elicited slower reactions than stimuli that were low or high on the subjective significance scale. The effect was particularly loaded by the group of highly arousing and moderately significant words, which evoked slower reactions than most other experimental conditions. Because we did not observe any behavioral effect of subjective significance in our current study, we conclude that the specificity of experimental stimuli may be responsible for the lack of replication of the earlier reaction time results (we used different word lists in the two experiments).^3^

The reason for a pattern of behavioral results incongruent with our previous findings, namely the valence effect caused by the neutral stimuli, the arousal effect in neutrally valenced conditions, and no subjective significance effect, may be based on methodological differences between this experiment and previous studies. In the stimuli selected for this study, the factor of valence was orthogonally crossed with arousal and subjective significance. In everyday life, valence is related to arousal in a quadratic way (i.e., negative and positive stimuli are far more arousing than neutral stimuli). When selecting valenced stimuli that are aligned in other properties, we have to search them more closely around the mean value for the scale; thus, their distribution has to be narrowed. In the current experiment, the mean valence for neutral stimuli was around 5 (on a 9-point Likert scale); for negative stimuli, it was around 4; and for positive stimuli, it was around 6 (see Supplementary Appendix 1). It is also possible that the simultaneous control of three manipulated and two controlled factors could have resulted in a list that biased to some extent the stimulus selection (narrowing the pool of available stimuli) and influenced the pattern of the results. In that case, the conclusion would be that the arousal level of neutral words is more important in generating a slowdown in responding than the arousal level in minimally (in an ecological sense) valenced stimuli, which should apply in general to verbal stimuli. It is possible that the valenced stimuli are typically associated with higher levels of arousal; therefore, participants could have framed them implicitly to other positive or negative states they experienced. Such a framing effect (c.f. Kahneman, 2011) would lead to the feeling that arousal is more silent and less intensive in emotional categories than in neutral ones.

The inclusion of subjective significance *per se* may also be the main reason for the pattern of results associated with valence. Researchers have found that subjective significance interacts with arousal in a way that can be summarized as the neutralization of high arousal slowdown in reaction times by the presence of subjective significance (both low and high). It is possible that valence itself enhances the subjective significance effects and results in a general reduction of reaction times in valenced conditions (c.f. the valence effect for highly arousing stimuli). This interpretation may also be supported by the replication of the ERP results found in an earlier study for the N450 component. Lastly, in the current experiment we used a different, more appropriate method of removing outliers (subject-wise) than in the previous studies, which could have influence on the results.

### Electrophysiological Results

#### Exploratory EEG Analysis

We observed that in the 250−350- and 350−520-ms time windows, the ERP amplitude was sensitive to the subjective significance of the stimuli. Specifically, the words of lower subjective significance evoked less positive amplitude than the words of moderate and high subjective significance, a finding that is congruent with our hypotheses. In our previous study (Imbir et al., 2017a), we showed that in a similar (290−530 ms) time window, there was a main effect of subjective significance based on the same pattern (i.e., the amplitude for stimuli of low subjective significance was less positive than for highly and medium significant stimuli). This effect was general and did not interact with specific ROIs in either previous or current studies. Thus, the results obtained in the series of studies seem to be consistent. We have interpreted the 290−530-ms time window as corresponding to the N450 component. According to van Hooff et al. (2008), the N450 component, occurring around 350–500 ms, is related to cognitive control and the mechanisms responsible for the suppression of conceptual representations. This pattern of results suggests that low subjectively significant conditions evoked the highest perceived conflict at this point of processing words, while other conditions were associated with lower conflict (Imbir et al., 2017a).

#### The EEG Results of Classical Components

In our study, we decided to analyze the results using a classical, component-based approach for two components found in our earlier studies (Imbir et al., 2017a, b) to be susceptible to cognitive control in the EST, namely P2 and N450. The time windows for the classical components P2 and N450 differed slightly from the time windows of exploratory analysis because when selecting them we based our choice on the literature (c.f. Luck, 2005; Citron, 2012) and on the obtained ERPs. For P2, we assumed 160–250 ms as the proper time range, while the N450 component time window was between 320 and 500 ms.

For the P2 component, we observed an interaction between valence and subjective significance, which is partially in line with our hypothesis. Further analysis of the interaction showed that effects were statistically significant mainly for neutral stimuli. The amplitude was more positive for medium subjectively significant than for both high and low subjectively significant stimuli. There was a clear effect of valence in the mentioned interaction within medium subjectively significant stimuli. The amplitude was considerably more positive for neutrally valenced stimuli than for the negative ones.

The amplitude of the P2 component is associated with a response to a threatening stimulus, usually highly arousing and negatively valenced (Thomas et al., 2007). In a previous study conducted by our team, we showed that the amplitude for highly arousing stimuli was larger than for moderately arousing ones (Imbir et al., 2017a), suggesting its role in interference control. Focusing on valence as a single variable, previous studies had shown contrasting results: larger amplitudes of P2 and shorter reaction times in response to negative rather than positive stimuli (Huang and Luo, 2006), in contrast to Schapkin et al. (2000), who reported larger amplitudes for positive rather than negative words in the P2 component. Some studies have also shown greater amplitudes for both positively and negatively valenced stimuli compared with neutral ones (Carretié et al., 2004; Herbert et al., 2006). Greater amplitudes for neutral words compared with the negative ones in the cluster of words medium on subjective significance in our study present yet another shape of differences regarding valence in the P2 component. On the other hand, this is consistent with behavioral results, where longer reaction times were observed in neutrally valenced incentives than in negatively valenced ones.

In the current study, we observed a subjective significance main effect within the N450 component. Low subjective significant incentives correlated with more negative amplitudes than those with a high and medium level of subjective significance. These outcomes are consistent with our hypothesis and previous studies, where a larger magnitude of the aforementioned component was observed for low significant stimuli than for moderately and highly significant words (Imbir et al., 2017a). We did not find the valence effect previously observed within this component (van Hooff et al., 2008), which could suggest that the factor of subjective significance was associated with valence.

#### Comparison Between Analytical Approaches

The comparison of analytical approaches gives us the opportunity to follow a timeline of an ERP and look into relations between proposed time windows. Chronologically, the first one – the classic P2 component, observed over frontal, central, and parietal parts of the scalp – revealed an interaction between valence and subjective significance. Nevertheless, when looking into particular differences between amplitude peaks for different conditions, one can see that only neutral words with a medium level of subjective significance evoked a potential that differed significantly from others. Controlling the level of arousal allowed us to remove the effect of emotional loads of words observed in other experiments (Schapkin et al., 2000; Carretié et al., 2004; Herbert et al., 2006; Huang and Luo, 2006). Consequently, the mutual control of valence and arousal may be responsible for not observing previously reported arousal effects, but the interaction observed in this study is similar to the one from our previous research (Imbir et al., 2017a). The dimension of subjective significance clearly has an impact on processing within the P2 component, which is also confirmed by studies employing self-related words (Fields and Kuperberg, 2012).

Following the timeline, one can see three effects, in two subsequent time windows and the classical N450 component, that are purely congruent: A low level of subjective significance evokes lower (more negative when the amplitude is below 0 and less positive otherwise) potentials than more subjectively significant words. The effects of the exploratory analyses were observed over the whole scalp, which indicates that it may influence the amplitude of a number of classical components with more specific localizations. The shape of the first exploratory-found ERP window (250−350 ms) led to negativity, but its peak was positive.

The subsequent effect is the continuation, where the ERPs observed over the whole scalp show a late (350–520 ms) negative peak. As these components are not observed in a particular ROI, it leaves some space for interpreting to which component or process it can be tied. We can consider the second exploratory-found time window as related to the N400 component. Effects of context were previously observed in this component, namely novel (incongruent) stimuli eliciting greater amplitudes than congruent ones (Kutas and Hillyard, 1984; Kutas, 1993; St. George et al., 1997; van Berkum et al., 1999; De Pascalis et al., 2009). If we interpret the factor of subjective significance as related to the novelty of the stimuli, we can conclude that the effect obtained in our study is congruent with these results: Words with low subjective significance – and thus less important and less known to the participant – elicited greater amplitude peaks than the highly significant, thus well-known ones. The relation between subjective significance and the novelty would need more exploration to confirm this conclusion.

The effects found in the exploratory-found time windows are certainly related to the subsequent N450 component, as the differences between the amplitudes in all three components have the same shape: Words with low subjective significance elicited lower amplitudes. This outcome is in line with our previous findings regarding subjective significance (Imbir et al., 2017a). The results found in the exploratory analysis need further research, because they suggest that the subjective significance may influence also earlier stages of processing, evoking differences in amplitudes observed over the whole scalp.

### General Discussion and Limitations

The main effect of valence in behavioral data was rather surprising, although it is possible that this effect is not an artifact (see the previous discussion of similar results in the literature and the role of inclusion of subjective significance). The mean reaction time for neutral words is mostly loaded by highly arousing words. As mentioned earlier in this article, arousal is tied to high intensity of the emotional experience. These results show that even for paradigms that have been used for decades, such as the EST, using newly proposed variables may reveal effects that could have been hidden from our view in previous studies due to a one-dimensional approach to emotional functioning.

The results of the current experiment are consistent with earlier EEG studies. The influence of subjective significance of the stimuli on their processing was observed along a very long part of an ERP, which is in accordance with our previous results (Imbir et al., 2017a). Specifically, we had observed the arousal effect in the P2 component, which, in the context of recent results, may be interpreted as being loaded by highly arousing neutral words, as valence was not controlled in the previous experiment. Highly arousing neutral words are a very specific cluster; thus, manipulating all three dimensions is a prominent advantage of this study. The stimuli that are low on subjective significance evoke lower potentials than any other stimuli for both the N450 component and the exploratory-found time windows (250−350 and 350–520 ms). The effect suggests that the cognitive control load was reduced by the increasing level of subjective significance, as predicted in the model of dual mechanisms of activation in the EST (Imbir, 2016b, c).

The study clearly has its limitations. With regard to the design, we highlight the number of manipulated and controlled dimensions of emotional processing. Because the large variance here may be explained by valence, arousal, and subjective significance, there is still space for exploring the effects of omitted dimensions, such as origin and concreteness (Siakaluk et al., 2014; Imbir et al., 2016). The dichotomic approach to the selection of the stimuli could also clarify some of the uncertainties regarding the observed effects; however, it may be considered as less carefully prepared, omitting the medium levels of emotional functioning and thereby violating the potential role of the Yerkes–Dodson laws (Imbir, 2017a). It is worth noting that three out of four reported ERP effects were in fact general, not connected to any particular region. Nevertheless, we found the most solid and repeatable effect for the localized N450 component.

## Conclusion

In the current study, we have demonstrated valence, arousal, and subjective significance effects on performance in the EST evidenced by the behavioral data as well as electrophysiological measures of ERP amplitudes in exploratory and classical approaches. We have only observed the behavioral slowdown in reaction times caused by arousal for neutral words. The exploratory ERP analysis revealed general subjective significance effects on the 250−350- and 350−520-ms time ranges, which may be interpreted as the N450 component observed slightly earlier and over the whole scalp. The classical confirmatory analysis showed indeed that the observed effects are specific to the N450 component. The current experiment replicated, with the use of another research manipulation (a broader list of words), the effect of subjective significance identified in an earlier experiment. In addition, the neutral words revealed the subjective significance effect in the P2 component, which was consistent with earlier studies. This suggests that subjective significance is an important factor to include, as well as valence, arousal, frequency of words, and their length, when interference control in the EST is considered.

## Data Availability Statement

The datasets presented in this study can be found in online repositories. The names of the repository/repositories and accession number(s) can be found below: https://figshare.com/s/d4d35d51286aabd4a8d4.

## Ethics Statement

The studies involving human participants were reviewed and approved by bioethical committee of the Faculty of Psychology at the University of Warsaw. The patients/participants provided their written informed consent to participate in this study.

## Author Contributions

All authors contributed to final version of the manuscript. KI: theoretical proposition. KI, MP, and MJ: introduction. KI and JŻ: design. KI, JD-G, and MP: method (words). JŻ and JD-G: method (EEG measures), experimental procedure programming, and experiment execution. JŻ, KI, JD-G, and MP: statistical analyses. JŻ, JD-G, and MP: results description. KI, MP, and MJ: results discussion. JD-G, JŻ, and KI: figures.

## Conflict of Interest

The authors declare that the research was conducted in the absence of any commercial or financial relationships that could be construed as a potential conflict of interest.

## Footnotes

1 In this study, similar to the current experiment, the verbal material was carefully selected. Words were contrasted in terms of types of activation mechanism (arousal versus subjective significance, both representing three increasing levels), while other factors, such as valence, concreteness, frequency, and word length (Kazojć, 2011), were controlled and aligned as moderate/neutral for all experimental conditions (Imbir, 2016c).

2 Using the filtfilt.m MATLAB function.

3 To validate this explanation, we decided to check whether words with different levels of subjective significance from both of the lists (two previous experiments using the same list of stimuli and the current one) that we have used in our studies differed significantly from the mean rating of subjective significance for the nouns from the ANPW-R study (Imbir, 2016a). We found that for both of the studies, the groups of subjective significance were ecologically accurate (i.e., representing different levels of the factor). One sample *t* test showed that the mean value of subjective significance from all the nouns in ANPW_R (M = 3.64, SD = 0.87) differed significantly from the groups of low (M = 2.92, SD = 0.40; *t*(44) = −12.03, *p* < 0.001) and high (M = 4.69, SD = 0.41; *t*(44) = 17.45, *p* < 0.001) subjective significance from the 2017 study, as well as from the groups of low (M = 3.01, SD = 0.28; *t*(134) = −26.38, *p* < 0.001) and high (M = 4.36, SD = 0.39; *t*(134) = 21.71, *p* < 0.001) subjective significance from the current study. The mean rating for moderately significant stimuli did not differ from the mean significance of the nouns from the ANPW-R database either for the 2017 study (M = 3.67, SD = 0.30; *t*(44) = 0.68, *p* = 0.5) or for the recent one (M = 3.62; SD = 0.14; *t*(134) = −1.38, *p* = 0.17). After ensuring that both of the stimuli sets were ecologically accurate, we verified whether the mean ratings of subjective significance for low, medium, and high significance groups differed between the two experiments. We used the Mann–Whitney *U* test because the groups consisted of a different number of words. We found significant differences in low significance groups between the cluster used in the 2017 experiment and the recent one (*U* = 2,184, *p* < 0.01): The words from the group in the current experiment had higher ratings, which can be interpreted as closer to moderate. The words from the high significance group used in the current experiment had lower ratings than those used in the 2017 experiment (*U* = 1,546, *p* < 0.001), which can also be interpreted as closer to moderate. The medium significance groups did not differ significantly (*U* = 2,817.5, *p* = 0.48). We conclude that the word cluster used in our experiment, regardless of being divided in a proper manner, had smaller differences between different levels of subjective significance than the one used in the previous study. This could be responsible for the lack of a behavioral effect of subjective significance: Because all the groups of words were closer to the medium values, we did not observe the medium-rated group standing out, evoking longer processing of the stimuli.
