# Supplementary material for: Event-Related Potential Correlates of Valence, Arousal, and Subjective Significance in Processing of an Emotional Stroop Task
Source: Front Hum Neurosci. 2021 Feb 25;15:617861. doi: 10.3389/fnhum.2021.617861 (PMC7947367; doi:10.3389/fnhum.2021.617861)
Supplement: Supplementary file 2 [file Table_2.DOCX]

Appendix 2


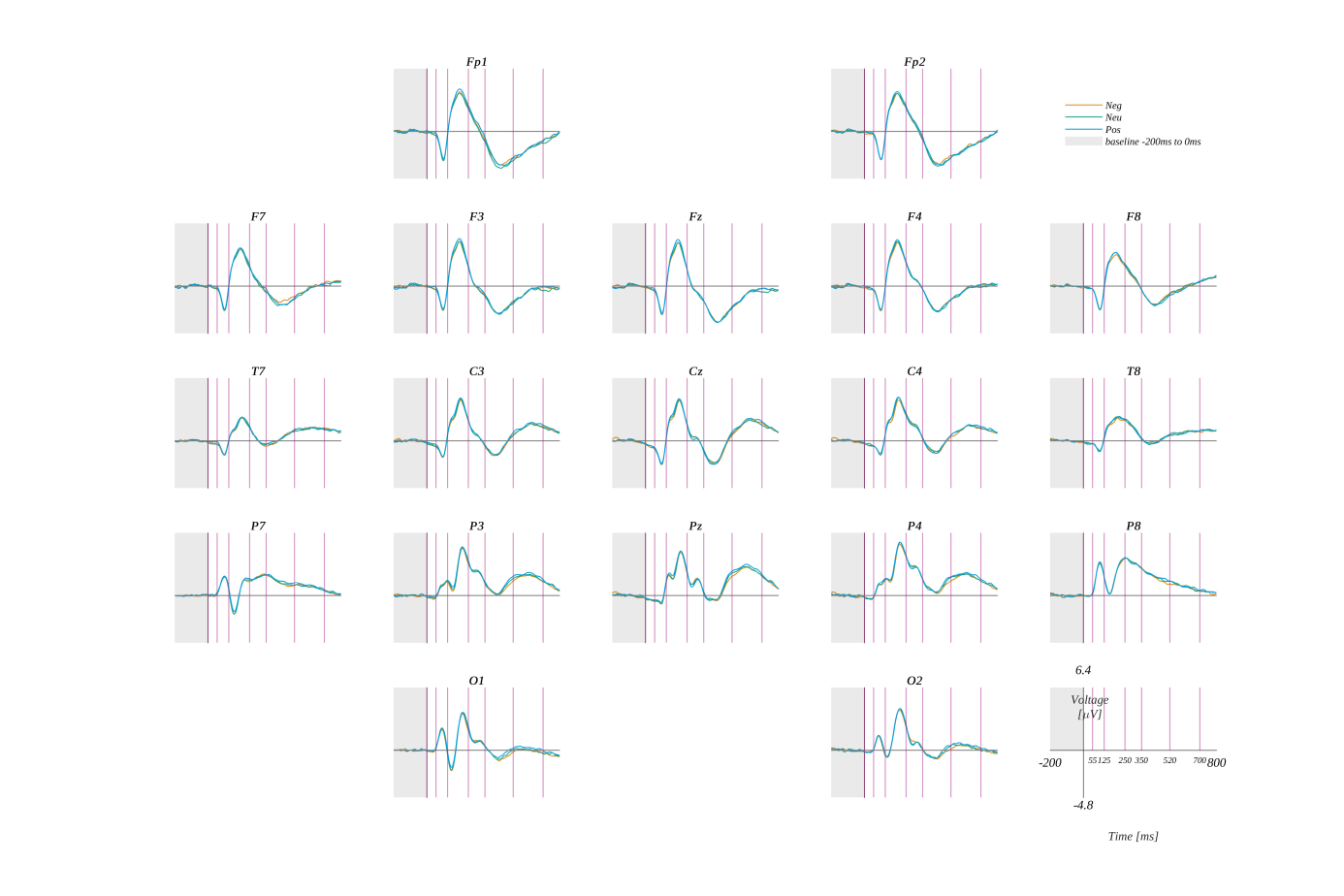


Figure A1. The time course of the ERP for levels of valence averaged across subjects. Gray rectangles highlight the time range selected for baseline corrections in subsequent analysis.


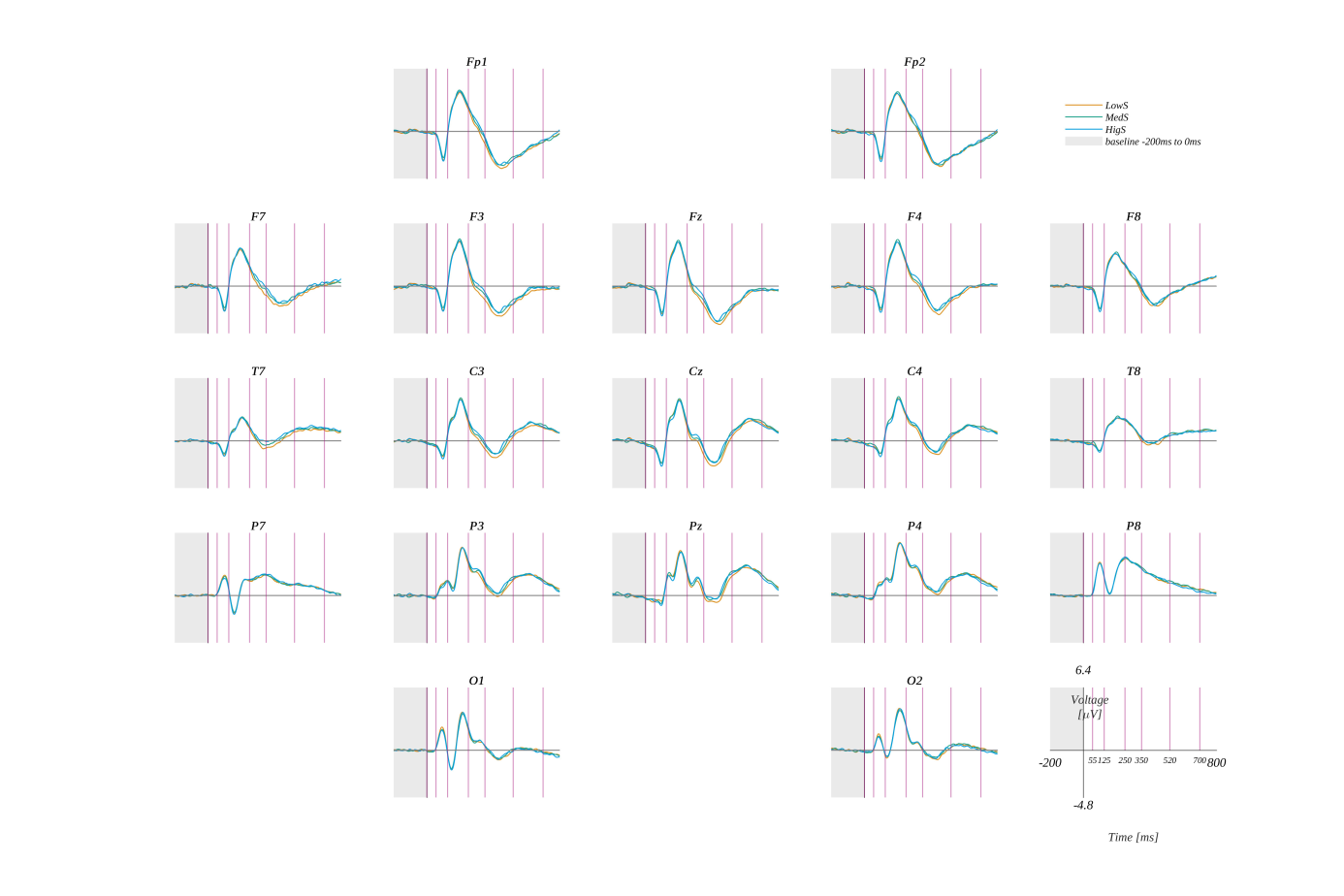


Figure A2. The time course of the ERP for levels of significance averaged across subjects. Gray rectangles highlight the time range selected for baseline corrections in subsequent analysis.


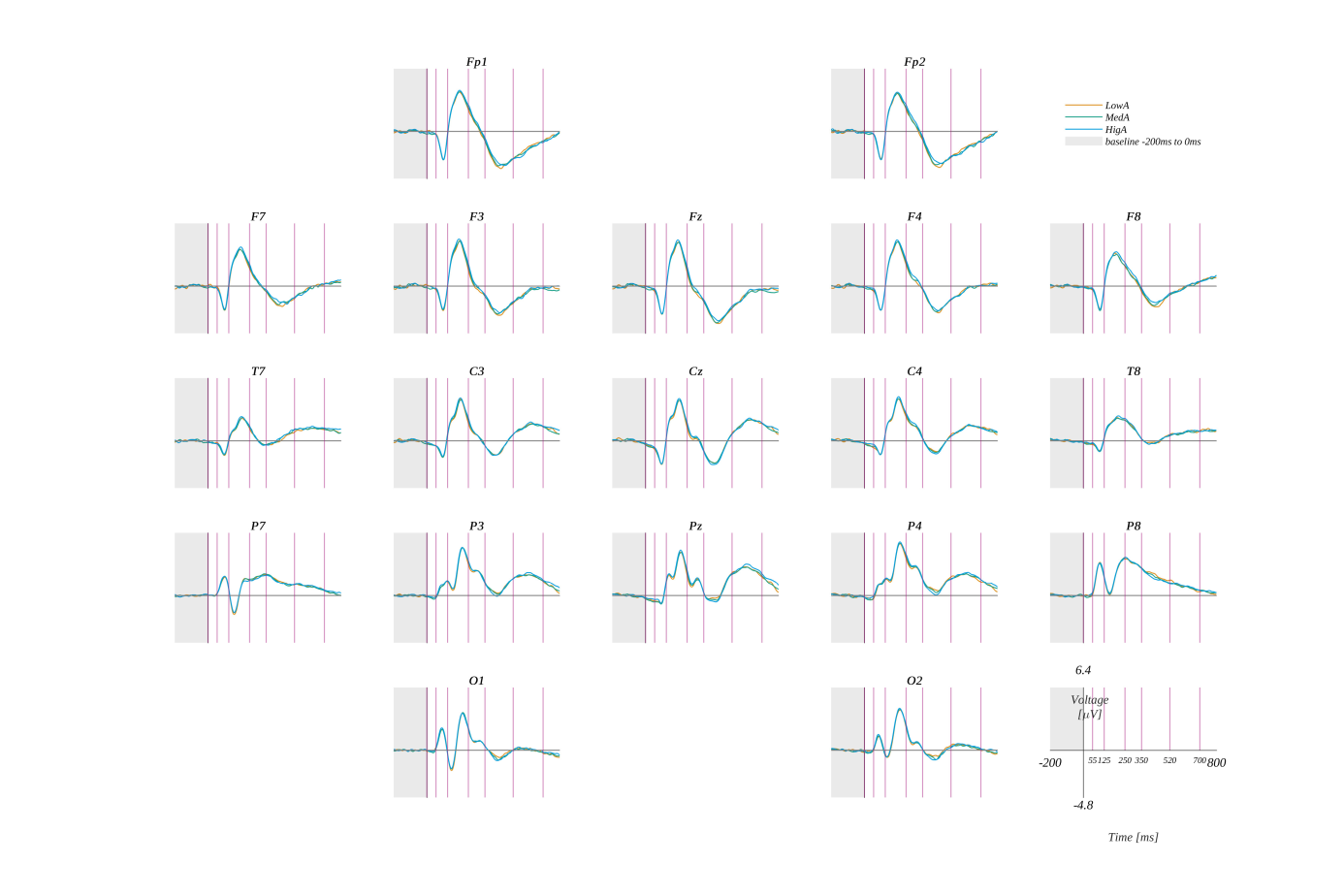


Figure A3. The time course of the ERP for levels of arousal averaged across subjects. Gray rectangles highlight the time range selected for baseline corrections in subsequent analysis.
